# A New Cellular Automaton Model for Urban Two-Way Road Networks

**DOI:** 10.1155/2014/685047

**Published:** 2014-11-04

**Authors:** Junqing Shi, Lin Cheng, Jiancheng Long, Yuanlin Liu

**Affiliations:** ^1^College of Engineering, Zhejiang Normal University, Jinhua 321004, China; ^2^School of Transportation, Southeast University, Nanjing 210096, China; ^3^School of Transportation Engineering, Hefei University of Technology, Hefei 230009, China; ^4^Wuhan Transportation Science Research Institute, Wuhan 430015, China

## Abstract

A new cellular automaton (CA) model is proposed to simulate traffic dynamics in urban two-way road network systems. The NaSch rule is adopted to represent vehicle movements on road sections. Two novel rules are proposed to move the vehicles in intersection areas, and an additional rule is developed to avoid the “gridlock” phenomenon. Simulation results show that the network fundamental diagram is very similar to that of road traffic flow. We found that the randomization probability and the maximum vehicle speed have significant impact on network traffic mobility for free-flow state. Their effect may be weak when the network is congested.

## 1. Introduction

Nowadays, traffic congestion has become a major and costly problem in many cities due to the growth of city population and vehicles. Developing simulation models for road traffic and discovering the fundamental laws of traffic dynamics can provide significant contributions to traffic congestion mitigation and prevention. In the past few decades, various models have been proposed to simulate traffic dynamics. Among them, cellular automata (CA) models have become more and more popular. This is because evolution rules of CA models are simple, straightforward, and efficient [1].

Since the one-dimensional CA traffic model (NaSch) [2] and the two-dimensional CA traffic model (BML) [3] were proposed in 1992, a great many CA models have been developed to simulate road traffic dynamics [4–20]. In 1999, a “unified” CA model of city traffic (Chsch) based on the NaSch model and the BML model was proposed [21]. So far, various factors have been considered into the CA models to enhance the ability of the models in simulating the metropolitan traffic phenomena [22–25]. However, most of existing models are developed for one-way traffic systems. In practice, two-way roads are more commonly found in urban traffic networks.

In this paper, a new CA model for urban two-way road networks is proposed. In our model, vehicles on roads directly follow the rules in the original NaSch model. To reduce vehicle conflicts and improve traffic efficiency, the vehicles in an intersection are assumed to have priority over the vehicles in the cells near the intersection. Two novel rules are proposed to move the vehicles in intersection areas, and an additional rule is developed to avoid the “gridlock” phenomenon. Simulations are carried out to investigate network fundamental diagram and the effect of the randomization probability and the maximum vehicle speed on network traffic mobility.

The rest of the paper is organized as follows. In Section 2, a new CA model is proposed for urban two-way road networks. In Section 3, simulation results are presented and discussed. Finally, conclusions are drawn in Section 4.

## 2. Model

**Figure 1:**
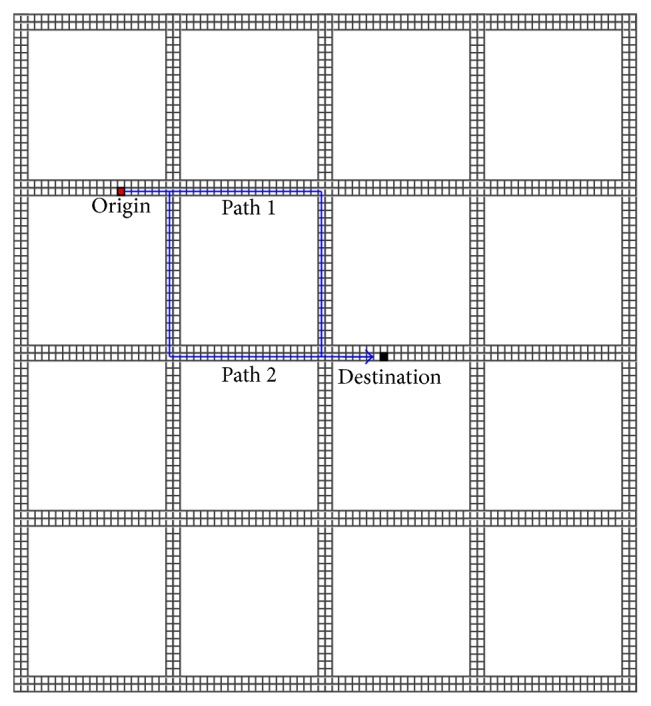
A two-way road network with *S* = 5 and *L* = 20.

As shown in Figure 1, an urban road network with *S* × *S* two-way roads is considered. Each road is divided into *L* cells, and the length of each cell is 7.5 m, and each car occupies one cell. Vehicles drive on the right-hand side of the road.

At the initial time, *N* cars are randomly distributed in the network. Each car is randomly assigned an origin and a destination. Beside the cells in intersections, all other cells can be taken as origins and destinations by cars. All cars are assumed to travel along the shortest path in terms of distance to their destinations. We adopt an additional distance to reflect the different impedance of each movement at intersections: 3, 1, and 2 cells for left turning, ahead, and right-turning movement, respectively. Then, the Dijkstra algorithm can be used to generate the shortest path tree, and each car randomly selects one shortest path to finish its travel. When a vehicle arrives at its destination, it will randomly select a new destination to continue its travel. Each car can do left turning, ahead, and right-turning movements at inner intersections but is not allowed to be driven in reverse on all roads.

The movement behavior of a car traveling through an intersection is quite different from that on a road. Hence, update rules of cars on roads and in intersection areas are separately described as follows.

### 2.1. Update Rules of Road Sections

**Figure 2:**
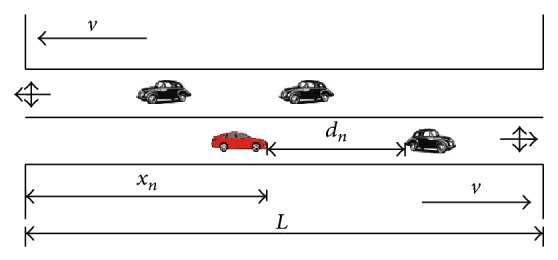
The sketch of road section.

The update rules of road sections directly follow the NaSch model [2]. Let *x*_*n*_ and *v*_*n*_, respectively, be the position and speed of the *n*th vehicle on a given road section (see Figure 2). Each vehicle has a maximum speed *v*_max⁡_, and *v*_*n*_ = 0,1,…, *v*_max⁡_. Then, *d*_*n*_ = *x*_*n*+1_ − *x*_*n*_ − 1 is the distance between the *n*th vehicle and the vehicle in front of it, and if the *n*th vehicle is the first vehicle, then *d*_*n*_ = *L* − *x*_*n*_. At each time step, the speed and position of each vehicle on a road section are updated in parallel according to the following rules.

**Step 1 (acceleration).**

If *v*_*n*_ < *v*_max⁡_, the speed of the *n*th vehicle is increased by one, but *v*_*n*_ remains unaltered if *v*_*n*_ = *v*_max⁡_; that is,

(1) $$\begin{array}{c} v_{n}⟶\min\left(v_{n}+1,v_{\max}\right). \end{array}$$

**Step 2 (deceleration).**

If *d*_*n*_ < *v*_*n*_, the speed of the *n*th vehicle is reduced to *d*_*n*_; that is,

(2) $$\begin{array}{c} v_{n}⟶\min\left(v_{n},d_{n}\right). \end{array}$$

**Step 3 (randomization).**

If *v*_*n*_ > 0, the speed of the *n*th vehicle is decreased randomly by unity with probability *P*; that is,

(3) $$\begin{array}{c} v_{n}⟶\max\left(v_{n}-1,0\right)\;\text{with}\;\;\text{probability}\;\;P. \end{array}$$

**Step 4 (vehicle movement).**

Each vehicle moves forward according to its new velocity determined by Steps 1–3; that is,

(4) $$\begin{array}{c} x_{n}⟶x_{n}+v_{n}. \end{array}$$

In Step 3, the randomization probability *P* is set to reflect the fact that vehicles may slow down due to some unpredictable factors, such as excessive brake, change of road conditions, psychological factors, and delay to accelerate. This probability can represent the effect of network environment on traffic flow.

### 2.2. Update Rules of the Vehicles in Intersection Areas

**Figure 3:**
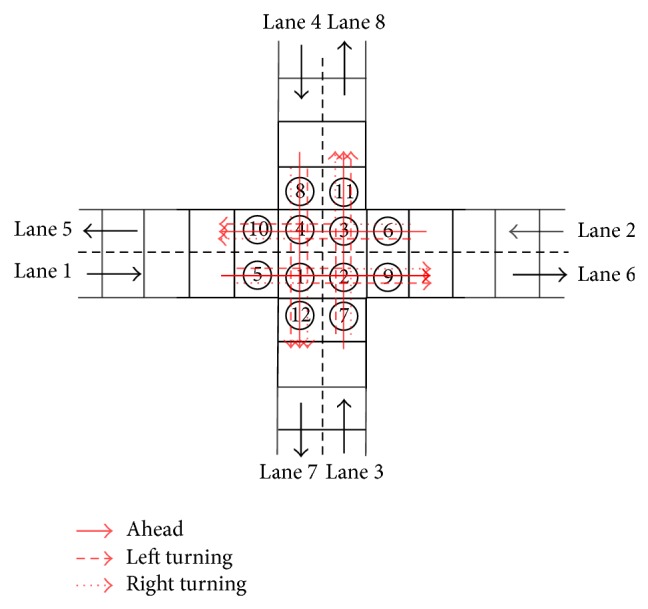
Cell representation of an intersection.

As shown in Figure 3, there are two types of cells related to each intersection: (i) cells in the intersection (i.e., Cells 1–4) and (ii) cells near the intersection (i.e., Cells 5–8). Vehicles of different directions travel through an intersection with different trajectories. For example, the left-turning vehicles on Lane 1 travel through Cells 5, 1, 2, 3, and 11 to Lane 8, ahead vehicles travel through Cells 5, 1, 2, and 9 to Lane 6, and right-turning vehicles travel through Cells 5, 1, and 12 to Lane 7. The remaining three directions follow the same movement pattern. We assume that the speed of a vehicle in an intersection is either 0 or 1. Hence, vehicles must travel through the cells on the trajectory in intersection areas one by one.

**Figure 4:**
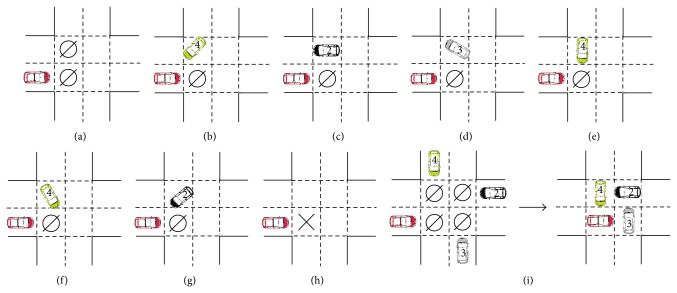
Update rules of the vehicles in intersection areas (e.g., take the vehicles from Lane 1). (a), (b), (c), and (d) show the four scenarios by which the vehicle is allowed to enter the intersection. (e), (f), (g), and (h) show the four occasions on which the vehicle is forbidden to enter the intersection. (i) shows the “gridlock” phenomenon. The number on the vehicle represents which lane it comes from. *⌀* represents that the cell is empty. × represents that the cell is occupied by a vehicle.

There are a total of 36 conflict points in each intersection and 9 conflict points for each cell in the intersection. To prevent vehicle collision, we assume that a vehicle in the cells in an intersection has priority over the vehicles in the cells near the intersection. For example, if Cell 4 is occupied by a left-turning vehicle from Lane 2 to Lane 7 or an ahead or left-turning vehicle from Lane 4, the vehicle in Cell 5 will be forbidden to drive into Cell 1. The following three rules will be adopted to update vehicles in intersection areas (see Figure 4).

*(i) Update Rules for Vehicles in Cells in the Intersection.* If the front cell is empty, then the vehicle moves forward one cell at the end of the step; otherwise, the vehicle will hold still. This rule will be adopted for all vehicles in Cells 1–4.

*(ii) Update Rules for Vehicles in Cells Near the Intersection. *If the front cell is empty and there are no vehicles in cells in the intersection attempting to occupy the cell, then the vehicle moves forward one cell at the end of the step; otherwise, the vehicle will hold still. This rule will be adopted for all vehicles in Cells 5–8.

*(iii) An Additional Rule for Vehicles Avoiding “Gridlock” Phenomenon*. We found that the “gridlock” phenomenon can occur for a special case: Cells 1–4 are empty, and Cells 5–8 are, respectively, occupied by an ahead or left-turning vehicle. In this case, if the four vehicles in Cells 5–8 simultaneously move forward one cell, then Cells 1–4 will all be occupied at the next step and the four vehicles can never move forward. To avoid the “gridlock” phenomenon, in such situation, we randomly select one vehicle in Cells 5–8 to hold still, and the other three vehicles move forward one cell.

## 3. Simulation Results

In this section, simulations based on the proposed CA model are carried out to investigate traffic characteristics in a two-way road network. The network size is 5 × 5 and the cell number of each road sections is 20 (i.e., 150 m). The network density is defined as the average number of vehicles that occupied one cell in the network. We varied the network density from 0.005 to 0.9 with an increment of 0.005. Ten times of simulations were carried out for each density. 20,000 time steps are simulated, and statistics are collected after 10,000 time steps of transient simulation. If the local deadlock happens before the end of simulation, the statistics are collected in accordance with the actual time steps of transient simulation.

### 3.1. The Network Fundamental Diagram

**Figure 5:**
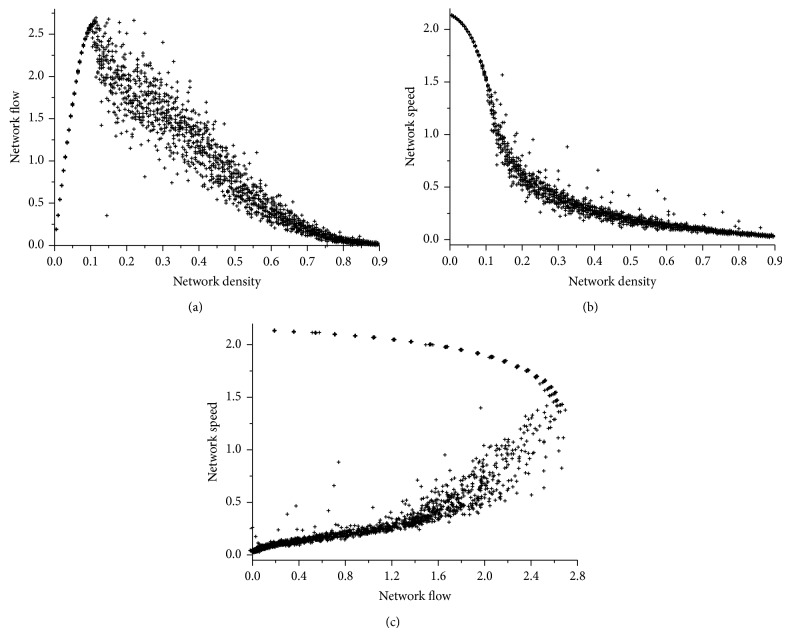
The network fundamental diagram: (a) flow-density relationship, (b) speed-density relationship, and (c) speed-flow relationship.

In a macroscopic traffic model, the fundamental diagram gives relations between traffic flow, density, and speed. It can be used to predict the capability of a road system or its behaviour when applying traffic controls. There also exists a fundamental diagram for the network traffic flow, which gives relations between network traffic flow, network vehicle density, and network speed. In this paper, network traffic flow is defined as the average number of vehicles arriving at destinations per unit time, and network velocity is defined as the average speed of the vehicles moving in the network. The network fundamental diagram is graphically displayed in Figure 5. One can observe that the corresponding relationships are very similar to that of road traffic flow.

- An approximate triangular fundamental diagram for network flow-density can be observed in Figure 5(a). The network traffic flow has a sustainable growth with network vehicle density, reaches its maximum value at a critical network vehicle density, and then drops gradually.
- Figure 5(b) shows that network speed drops gradually as network vehicle density grows up. The fundamental diagram for network speed-density has an inverse “S” sharp. This result is consistent with the fact that a more congested network has lower network speed.
- The network speed-flow relationship is not a one to one mapping. There are two network speeds corresponding to every network flow except the maximum network flow (see Figure 5(c)). One of the two network speeds indicates a free-flow state, and the other indicates a congested state.

### 3.2. The Effect of the Randomization Probability

**Figure 6:**
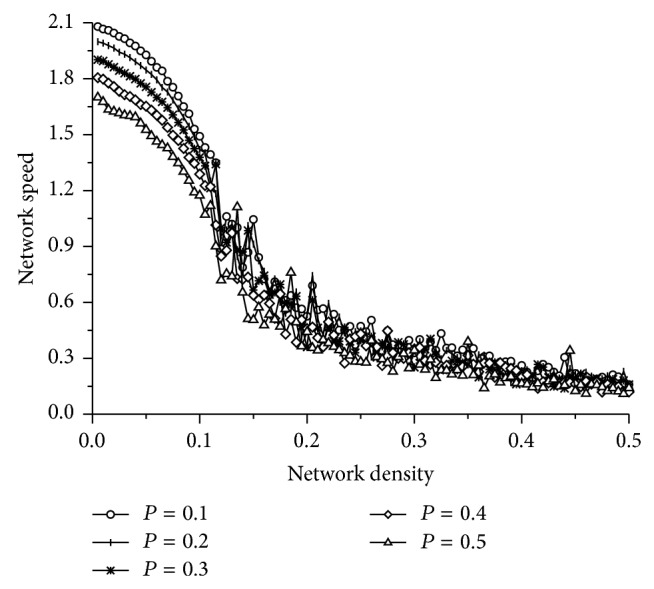
The influence of the randomization probability *P*.

The influence of the randomization probability on network traffic flow is graphically displayed in Figure 6. One can observe that the network speed is greatly influenced by the randomization probability when the network density is lower than a critical density. However, the influence will be weak when the network density exceeds the critical density. If the network density is lower than the critical density, a lower randomization probability can bring a higher network speed. This is because vehicles can move freely when the network density is low, and the vehicles are more likely to keep a high speed with a small randomization probability. When the network density is larger than the critical density, vehicles may frequently be in a state of stop-and-go, and the influence of the randomization probability disappears.

### 3.3. The Effect of the Maximum Vehicle Speed

**Figure 7:**
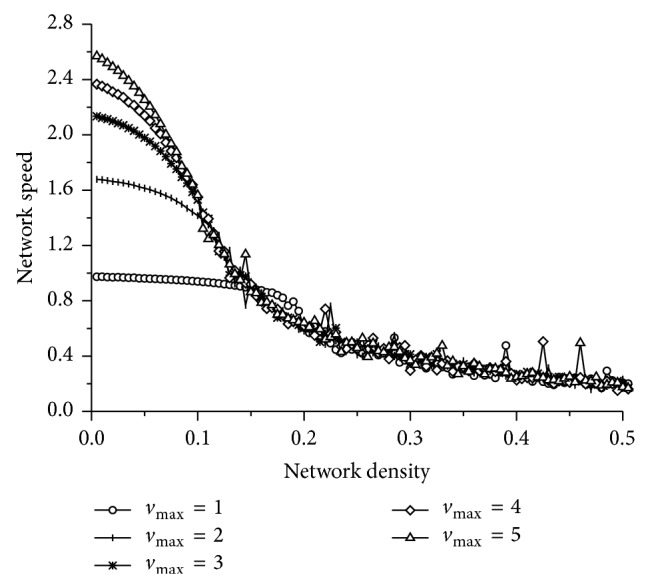
The influence of the maximum speed on network speed.

The influence of the maximum speed *v*_max⁡_ on network traffic flow is graphically displayed in Figure 7. One can observe that the network speed is greatly influenced by the maximum vehicle speed when the network density is lower than a critical density. However, the influence will be weak when the network density exceeds the critical density. If the network density is lower than the critical density, a higher maximum vehicle speed can bring a higher network speed. This is because vehicles can move freely when the network density is low, and the vehicles are more likely to drive in a high speed. When the network density is larger than the critical density, vehicles cannot speed up due to traffic congestion, and the influence of the maximum vehicle speed disappears.

## 4. Conclusion

In this paper, a new cellular automaton model for urban two-way road networks was proposed. The simulation results showed that the network fundamental diagram of the network traffic flow is very similar to that of road traffic flow. We also found that both the randomization probability and the maximum vehicle speed can significantly influence traffic efficiency of networks with low vehicle density. Their influence will be weak when the network becomes congested. In the future, we will consider traffic flow control for the two-way network systems, such as signal control [26], information guidance [24], and vehicle movements bans [27–29].
